# Epidemiological characteristics of human infections with avian influenza A(H5N6) virus, China and Laos: A multiple case descriptive analysis, February 2014–June 2023

**DOI:** 10.14745/ccdr.v50i12a09

**Published:** 2024-01-01

**Authors:** Simran Sandhu, Christina Ferrante, Aaron MacCosham, Nicole Atchessi, Christina Bancej

**Affiliations:** 1 Centre for Emerging and Respiratory Infections and Pandemic Preparedness, Public Health Agency of Canada, Ottawa, ON

**Keywords:** influenza A virus, H5N6 subtype, pandemics, prevalence, China, Laos, influenza, human

## Abstract

**Background:**

The first human infection with highly pathogenic avian influenza A(H5N6) virus was reported in 2014. From then until June 30, 2023, 85 human cases with confirmed A(H5N6) infection have been reported worldwide.

**Objective:**

To address the present gap in knowledge of the overall epidemiology of human A(H5N6) infections, the epidemiological characteristics of human infection with A(H5N6) in China from February 2014 to June 2023 are described.

**Methods:**

Considering the severity of human infections with A(H5N6) virus (case fatality rate: 39%), the increased frequency of case reports from 2021 to present day, and lack of comprehensive epidemiologic analysis of all cases, we conducted a multiple-case descriptive analysis and a literature review to create an epidemiologic profile of reported human cases. Case data was obtained via a literature search and using official intelligence sources captured by the Public Health Agency of Canada’s International Monitoring and Assessment Tool (IMAT), including Event Information Site posts from the World Health Organization.

**Results:**

Most human A(H5N6) cases have been reported from China (China: 84; Laos: 1), with severe health outcomes, including hospitalization and death, reported among at-risk populations. The majority (84%) of cases reported contact with birds prior to illness onset. Cases were detected throughout the course of the year, with a slight decrease in illness incidence in the warmer months.

**Conclusion:**

As A(H5N6) continues to circulate and cause severe illness, surveillance and prompt information sharing is important for creating and implementing effective public health measures to reduce the likelihood of additional human infections.

## Introduction

Avian influenza A(H5N6) is a highly pathogenic avian influenza (HPAI) reassortant virus ((1,2)). Waterfowl are a common reservoir for avian influenza viruses, including A(H5N6) (3)). Transmission of A(H5N6) among birds can occur via infected secretions and droppings, and asymptomatic transmission of A(H5N6) among some wild bird species has been previously documented (4). Even though A(H5N6) mainly infects birds, humans have also been infected by the HPAI virus through zoonotic transmission of the virus. Humans can be exposed to A(H5N6) by both direct and indirect contact with infected poultry or contaminated environments. For example, a notable risk factor for human exposure to A(H5N6) and other avian influenza viruses are live poultry feeding and trading markets ((5)). When human cases of A(H5N6) are detected, these cases are reportable to the World Health Organization (WHO) under the International Health Regulations (2005) ((6)).

Severe disease and high mortality are often found in association with A(H5N6) infections among both animal and human populations ((7,8)). In Asia in 2014, A(H5N6) was first detected in domestic and wild bird populations. Since 2014, outbreaks have continued to be reported in bird populations worldwide. Detections have been reported to the World Organisation for Animal Health (WOAH) from 21 different countries in Asia, Europe, and Africa by the end of 2021. In 2022, an A(H5N6)-infected bird was reported in a 22^nd^ country, Canada, marking the first such detection in the Americas. This event highlighted the spread of A(H5N6) virus in the animal population and the increased risk of exposure, and thus infection, in humans. As of June 30, 2023, 29 different countries have reported detections of A(H5N6) in animal or bird populations since 2014 (7,8).

Considering the prevalence of A(H5N6) in birds globally, the diversity of currently circulating avian influenza viruses (AIVs), and interactions between host species, conditions could be favourable for reassortment and continued zoonotic transmission ((9,10)). The earliest detection of a human case of A(H5N6) was in a poultry dealer from Sichuan Province, China in 2014, soon after outbreaks of A(H5N6) were initially reported in birds in Laos, China, and Vietnam ((2,11)). This fatal case had occupational exposure to poultry prior to illness onset. Human cases of A(H5N6) have continued to be reported every year since, with a marked increase in detections in 2021 ((12)). The diversity of circulating AIVs, along with continued interaction between host species, can allow for continued reassortment and transmission of A(H5N6) ((9,10)). Therefore, the reported increasing prevalence of A(H5N6) in bird populations may be related to the increase in human cases.

To the best of our knowledge, no recent study has presented the epidemiologic characteristics of a comprehensive group of human A(H5N6) infections. Previously assessed studies were either written as case reports or only included a select subset of cases, leaving a gap in knowledge of the overall epidemiology of human A(H5N6) infections ((13–17)). This study aims to address this gap by summarizing the epidemiology of reported laboratory-confirmed human cases of A(H5N6) with illness onset dates from February 2014 to June 30, 2023. Building a better understanding of human infections with A(H5N6) is key for the consideration of modern public health measures that may help mitigate AIV A(H5N6) disease transmission.

## Methods

### Search strategy and selection criteria

A literature review on the epidemiology of A(H5N6) infections in the human population was conducted. The literature review involved both published and grey literature, including primary studies, commentaries, and reviews that assessed the human epidemiology of A(H5N6), reviews of animal studies to provide an overview of what is currently known about reported animal infections with A(H5N6), and reports from well-recognized public health authorities like the WHO, WOAH, the European Centre for Disease Prevention and Control (ECDC), the United States Centers for Disease Control and Prevention (CDC), the Government of the Hong Kong Centre for Health Protection (CHP), and submissions from national laboratories to the Global Initiative on Sharing All Influenza Data (GISAID). The Public Health Agency of Canada’s International Monitoring and Assessment Tool (IMAT) was also used to identify human cases of A(H5N6) and associated case information. The IMAT is a database that enables systematic documentation of information from event-based and other intelligence sources, as well as event verification and assessment of human emerging respiratory pathogens through official government sources. Trained epidemiologists conduct daily monitoring of these event-based surveillance sources and enter events as completely as possible into the IMAT using a standardized data capture form. These events are maintained and updated in the IMAT if or when more complete information becomes available from these sources.

**Figure 1:**
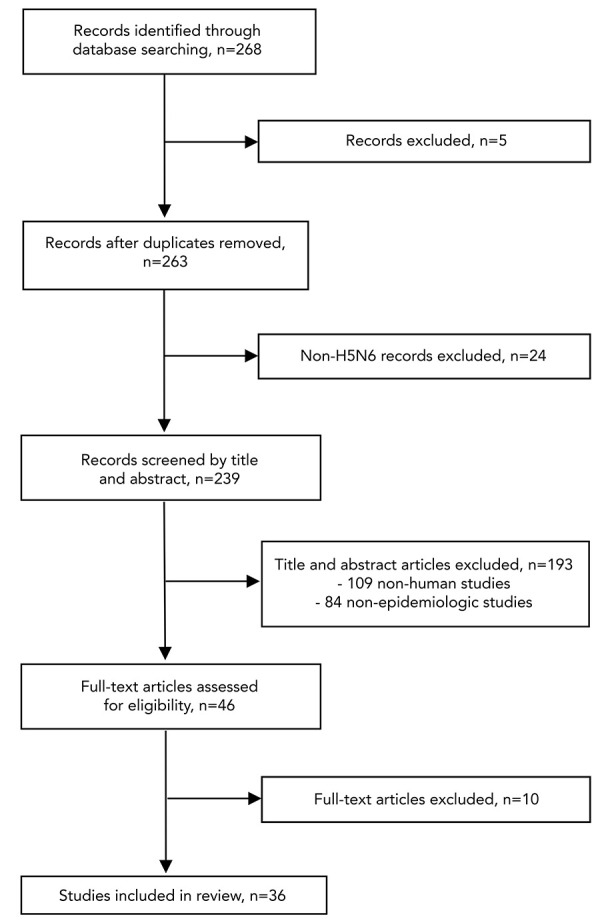
Flow diagram for study inclusion and exclusion in systematic literature review of the epidemiology of A(H5N6) in the human population

The literature search, conducted by a Health Canada Librarian, contained research published up until October 6, 2021, in the following databases: Ovid MEDLINE^®^ and Epub Ahead of Print, In-Process, In-Data-Review & Other Non-Indexed Citations, Daily and Versions^®^, Embase, Global Health, and CAB Abstracts. Literature in both English and French was sought, with the following search terms specified to the time period of relevance: H5N6, AH5N6, A?H5N6, A H5N6, and influenza virus A H5N6. Following this literature search, pre-specified screening criteria were used to identify studies for inclusion in data extraction and synthesis (**Figure 1**). Screening exclusion criteria included: non-translated materials in a language other than English or French, duplications missed in the initial literature search, non-H5N6 records, non-human and/or non-epidemiology primary studies, publications with unretrievable full texts, and studies outside our predefined themes of interest (epidemiology, microbiology/virology/genomics, diagnostics/testing, vaccines/therapeutics, public health measures/response, and risk assessment). Independent reviewers screened citations in duplicate and reached a consensus on which materials to include after discussion of any conflicts. In total, 36 published studies were eligible for inclusion. During the study period, reports for new human cases of A(H5N6) were also reviewed and data was extracted from both grey literature (via the IMAT) and published literature. For cases of A(H5N6) identified after October 6, 2021, the IMAT’s intelligence sources were used to assess and validate information about human cases of A(H5N6) that had a symptom onset date (or report date where symptom onset date was not available) after October 6, 2021.

### Case definition

Human cases of A(H5N6) were considered to be those reported by the WHO through its Event Information Site (EIS) posts or those reported by Government of Hong Kong’s CHP via an official publication. As the literature search was conducted, several journal articles that also referenced human A(H5N6) cases were cross-checked with the data available from official sources for validity and then included in the case line list. These referenced human A(H5N6) cases were either reported as or assumed to be laboratory-confirmed.

No standard case definition for human cases of A(H5N6) currently exists. The current WHO case definition for human cases of non-seasonal influenza are individuals with laboratory confirmation of a recent infection with non-seasonal influenza virus in a person, where the infection has been confirmed by positive results from the polymerase chain reaction (PCR), virus isolation, or paired acute and convalescent serological tests ((18)). This definition can be adapted to various non-seasonal influenza viruses, including A(H5N6). At this time, it is unclear if Government of Hong Kong’s CHP case definition for human cases of A(H5N6) differs from the WHO’s case definition.

### Data elements and extraction

A case identification number (case ID) was assigned to each case and data was extracted for various administrative, demographic, exposure, course of illness, and outcome data elements such as report date, age, sex, occupation, comorbidities, reporting geographic regions, animal contact history, symptoms, symptom onset date, hospitalization status, hospitalization date, discharge date, current disposition, outcome, and outcome date. Information corresponding to the data elements for each case was input manually into a line list using Microsoft Excel. Whenever conflicting information pertaining to various extracted data elements was presented by different sources of information, the validity of the source was relied upon to determine which data to extract. For example, a publication by the WHO took precedence over journal articles because the data contained in the WHO EIS posts contain information from official and confirmed government sources.

### Analysis

Descriptive analyses included all laboratory-confirmed human cases of A(H5N6) reported by symptom onset date from 2014 to June 30, 2023 (n=85). The descriptive analyses consisted of the calculation of median age and age range for cases, gender proportion, exposure proportion by age group, types of exposure sources, geographic distribution of cases, disease severity, and case outcomes. For all cases with available data, the main descriptive analysis consisted of seven variables: 1) median age; 2) age range; 3) proportion of males; 4) proportion hospitalized; 5) median hospitalization delay (days); 6) hospitalization delay (range); and 7) case fatality rate (CFR). The descriptive analyses were also stratified by sex: 1) male; 2) female; and by age group: 1) children (younger than 18 years); 2) adults (18 years or older). Case exposure source was also analyzed for cases with available exposure data.

Case data was analyzed by time period to have a better understanding of the characteristics and reported case incidence from 2021 to June 30, 2023, since there was a large increase in reported human A(H5N6) cases during 2021. For this analysis, the number of reported cases and geographic regions in which cases were reported were described by year, based on symptom onset or report date. Median age and age range for the cases, sex of cases, and case outcomes were described using Microsoft Excel.

Case data was also analyzed by season to understand if there is seasonality associated with reported human cases of A(H5N6), as has been suggested in the literature. For this analysis, months of the year were grouped into four seasons: 1) Spring: March, April, May; 2) Summer: June, July, August; 3) Fall: September, October, November; and 4) Winter: December, January, February. Reported human cases were then categorized according to symptom onset date or report date where symptom onset date was not available (n=1).

To assess disease severity, cases were analyzed by outcome, for which three variables were used: 1) survived; 2) deceased; and 3) unknown. For each category, the median case age and the age range, the proportion of cases for each category that were male, the proportion of cases hospitalized per outcome, the median and range of hospitalization delay (in days) per outcome, and the proportion of cases that were critically ill at the last known disposition for each outcome were calculated using Microsoft Excel. Where hospitalization status and last known disposition of cases was unknown, these cases were removed from the analysis where this data was required.

Case data was also described by geographic location of the reported case. Geographic locations were extracted from case reports and pertinent articles from the literature search. Based on reported province, cases were assigned to their respective province. Cases were then stratified by symptom onset date or report date where symptom onset date was not available and summed. This analysis was conducted using Microsoft Excel. The figure depicting geographic distribution was created using RStudio.

Data manipulation and analyses were conducted using RStudio and Microsoft Excel 2016 software. No cases were dropped from the analytic dataset. Where case details required for a specific analysis were missing, these cases were dropped from the particular analysis. Symptom onset date was unavailable for one case, so where symptom onset date was required for the analysis, report date was used for this case instead.

## Results

### Demographic characteristics

A total of 85 human cases of A(H5N6) were reported from two countries worldwide from February 2014 to the end of June 2023 (**Table 1**). Thirteen of these cases were identified retrospectively from non-surveillance sources, such as research articles. The median age of these cases was 50 years, with an age range of 1–81 years. Thirteen (13/85; 15%) reported cases were children younger than 18 years of age. Approximately half (46/85; 54%) of the cases were male. Out of the cases with known exposure data (71/71; 100%), all reported contact with birds prior to illness onset. Contact methods included visiting live bird markets (LBMs), contact with or employment as poultry workers, and exposure to slain and cooked poultry and/or domestic or backyard poultry. Thirty-one cases reported occupational background, and the majority of these cases (22/31; 71%) were either farmers, dealers with LBM contact, or slaughterhouse workers, all of which are professions with obvious potential for poultry exposure.

**Table 1:** Distribution of human cases of A(H5N6) by country, February 1, 2014 to June 30, 2023

Country	Symptom onset date of first case	Symptom onset date of latest case	Number of reported cases	Number of reported deaths
China	2014-02-16	2023-05-19	84	33
Laos	2021-02-28	2021-02-28	1	0

### Timeline and seasonality

On May 5, 2014, China reported one fatal A(H5N6) case from Sichuan Province, marking the first official report of a human A(H5N6) infection. However, an even earlier case was identified by researchers retrospectively, and case details published in a journal article (17). This case was a child who developed symptoms on February 16, 2014 (17). The case was identified in Hunan Province, a region that has reported nearly one fifth of all human cases to date (15/85; 18%) and the second-highest number of human cases total.

**Figure 2:**
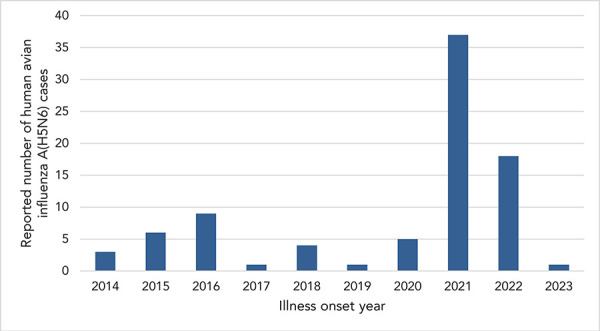
Epidemiologic curve of reported human cases of A(H5N6) by year of symptom onset, February 1, 2014 to June 30, 2023 (n=85)

**Figure 3:**
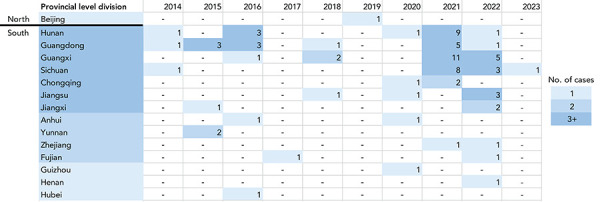
Geographic spread of human cases of avian influenza A(H5N6) in China by year of illness onset or report date, February 1, 2014 to June 30, 2023 (n=84)

**Figure 4:**
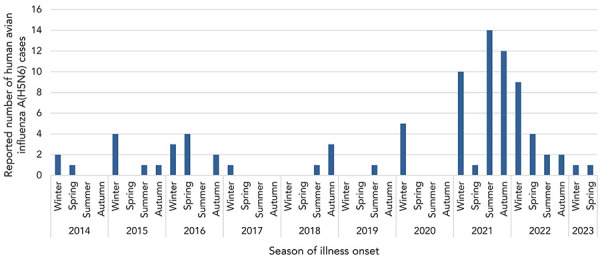
Epidemiologic curve of human avian influenza A(H5N6) infections, by season of illness onset date, February 1, 2014 to June 30, 2023 (n=85)

In 2021, a spike in case incidence was observed, with 37 cases (37/78; 47%) reporting symptom onset in this same year (**Figure 2**). The cases reported in 2021 from China were detected from six different regions in comparison to a median of three different regions annually in previous years (range: 1–5) (**Figure 3**). Furthermore, 2021 was the first year a human A(H5N6) case was detected outside of China. The cases with illness onset in 2021 had a similar profile to cases reported earlier: their median age was 54 years (range: 3–79) and 59% of the cases (22/37) were males. Outcome data were available for 15 of the cases from 2021 and indicated an annual CFR of 80% (12/15). Compared to 2021, fewer human cases with illness onset were reported in 2022 (18 cases), with a median age of 59 years (range: 3–68) and a similar sex distribution (13/18; 72% males). Outcome data were available for two cases from 2022, of which both cases (2/2; 100%) were fatalities. Human cases of A(H5N6) continue to be reported into 2023, and as of June 30, 2023, one case reported illness onset this year. Study results indicate that cases are detected throughout the course of the year, with a slight decrease in illness incidence into the spring and summer (**Figure 4**).

### Geographic distribution

**Figure 5:**
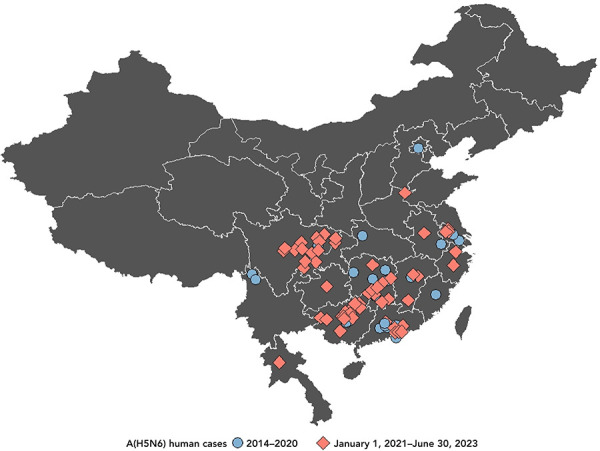
Spatial distribution of human cases of avian influenza A(H5N6) in China and Laos, February 1, 2014 to June 30, 2023 (n=85)

Almost all (84/85; 99%) human A(H5N6) infections have been reported from China, from 15 different regions: Guangxi Zhuang Autonomous Region (19 cases), Hunan Province (15 cases), Guangdong Province (14 cases), Sichuan Province (13 cases), Jiangsu Province (five cases), Chongqing Municipality (three cases), Jiangxi Province (three cases), Anhui Province (two cases), Yunnan Province (two cases), Fujian Province (two cases), Zhejiang Province (two cases), Beijing Municipality (one case), Guizhou Province (one case), Hubei Province (one case), and Henan Province (one case) (**Figure 5**). The majority of these cases were concentrated in south or southeast China, areas that have a high density and popularity of LBMs and free-range farming practices, and also areas rich in water resources that are habitats for AIV hosts ((19)). Geographic spread seems to be occurring, with not only the number of regions from which cases are reported from China increasing, but also with one case detected in northern China for the first time in August 2019 and one case reported from a bordering country, Laos, in March 2021 ((16)) (Figure 3).

### Disease severity

Human cases of A(H5N6) have a clinical manifestation similar to human infections with other HPAI H5 viruses: symptoms often begin with fever, upper respiratory tract symptoms, and myalgia. Soon afterwards, a rapid progression to lower respiratory tract illness often results in pneumonia, multiple organ failure, acute respiratory distress syndrome (ARDs), and death ((20)). At least 33 case fatalities have been reported overall (CFR: 33/85; 39%) ((21)) and of the cases with unknown outcome but available disposition data, 90% (36/40) were in critical or severe condition at the time of the last report (**Table 2**). Among the 80 cases with available hospitalization data, 95% (76/80) required hospital admittance and of the 68 cases with available hospitalization and symptom onset dates, 91% (62/68) were admitted within one week (seven days) of illness onset (median hospitalization delay: four days; range: 0–13 days), further highlighting the severity of this disease (**Table 3**). Intensive care unit (ICU) admission details were too sparse to draw conclusions.

**Table 2:** Clinical characteristics of laboratory-confirmed human cases of avian influenza A(H5N6) by outcome status, February 1, 2014 to June 30, 2023

Outcome	Age (years)		Proportion male	Proportion hospitalized (n=78)	Hospitalization delay		Proportion critically ill at last known disposition^a^ (n=39)
	median	range	%	%	days^b^	range	%
Survived (n=14)	31	1–65	36	71	3	0–10	14
Deceased (n=27)	49	3–81	48	100	5	0–13	N/A
Unknown (n=44)	53	3–72	64	100	3	0–10	90

Abbreviation: N/A, not applicable

^a^ Proportion critically ill at last known disposition means the proportion of cases that were reported as critically ill (instead of stable, alive, or deceased) at the time of the last report, or at the time of the last report prior to final outcome

^b^ Hospitalization delay (days) means time between symptom onset date and hospitalization date, in days

**Table 3:** Descriptive characteristics of human cases of A(H5N6) by sex and age groups, February 1, 2014 to June 30, 2023

Variable	All cases (n=85)	Sex		Age group	
		Male (n=46)	Female (n=39)	Children (<18 years) (n=13)	Adults (≥18 years) (n=72)
Median age (range)	51 (1–81)	51 (3–79)	47 (1–81)	5 (1–12)	52 (22–81)
Proportion males (%)	54	N/A	N/A	18	58
Proportion hospitalized (%)	95	98	92	69	100
Median hospitalization delay (days)^a^ (range)	4 (0–13)	4 (0–13)	3 (0–10)	5 (1–10)	3 (0–13)
Case fatality rate (%)	39	72	61	44	72

Abbreviation: N/A, not applicable

^a^ Hospitalization delay (days) means time between symptom onset date and hospitalization date, in days

Outcome data were only available for 48% of the cases (41/85), and of these individuals, two thirds (27/41; 66%) died. In comparison to nonfatal cases, fatal cases were older, had a higher proportion of males and hospitalizations, and experienced a greater delay in hospitalizations (Table 2). Of the fatal cases with known comorbidity information, half (3/6) reported the presence of comorbidities, as opposed to one third (1/3; 33%) of the non-fatal cases. However, comorbidity information was very infrequently reported.

## Discussion

Avian influenza A(H5N6) remains a deadly virus, having killed approximately four out of every ten reported human cases (Table 3). Most of these cases, including those that survived, were severe and required hospitalization (Table 2 and Table 3). Existing evidence from serological studies of humans at high risk of exposure also suggests mild or asymptomatic illness is uncommon with A(H5N6) and less likely as compared with other AIVs ((19)). To date, 85 human cases of A(H5N6) have been reported worldwide, mostly from south or southeast China, but geographic spread is present with cases reported from other regions and Laos in recent years. Human infections have continually been reported since the emergence event in 2014, with an increase in cases in 2021 (Figure 2). It is possible that this increase in cases coincides with heightened surveillance and diagnostic systems resulting from the coronavirus disease 2019 (COVID-19) pandemic, but other factors, like the spread of AIVs in poultry populations, likely also play a role in the increased number of cases since most cases seem to be infected post-exposure to infected poultry or contaminated environments. Regardless, this increase in cases serves as a reminder that the epidemiology of human AIV infections may change at any time due to the transformative nature of these viruses. This further emphasizes the need to continue surveillance and situational assessments of human infections with AIVs. Each event should be scrutinized for changes that may result in increased infectivity or pathogenesis. In general, an increase in reported cases was observed in cooler seasons (Figure 4). These study results corroborate literature postulating an increased incidence in the winter and autumn months, coinciding with influenza A seasonality in humans and aligning with avian migratory pathways ((22)). The majority of cases were middle-aged, with a median age of 51 years, but viral infections have been reported in children and seniors as well (Table 3). Both sexes seem equally susceptible to infection (Table 3). Although avian influenza A(H5N6) rarely infects humans, certain populations are at an elevated risk of infection, such as those with exposure to birds. In the past, human A(H5N6) cases have been linked to local live poultry feeding and trading markets. through genetic analysis and comparison of viral case and environmental samples ((21)). Epidemiological investigations have also revealed positive H5 results from the backyards of several cases in China who kept domestic poultry or had wild birds frequent their residences (21). Workers with occupational exposures, such as poultry sellers, are also at higher risk of positive serology and investigators have observed positive A(H5N6) serology specimens from poultry workers in the past, although this does not constitute a positive case. Since A(H5N6) is transmitted through secretions and droppings, exposure to birds in these environments may increase risk of infection through direct or indirect contact with infected poultry.

China is considered a hotspot for the emergence and spread of AIVs due to their widespread persistence, the well-established and growing poultry production and trading industry, and the mixing of host species in live bird markets ((23–25)). To mitigate the risk of animal-to-person transmission, the appropriate use of personal protective equipment is vital, and other biosecurity and preventive measures, such as antiviral prophylaxis after potential exposure, should be used as safeguards where applicable ((26)). Adhering to public health measures like regular thorough handwashing, staying home if feeling sick, and minimizing contact with wild, sick, and/or dead birds and contaminated and/or high-risk environments like LBMs may protect individuals from A(H5N6) infection. Seasonal influenza vaccination may also help prevent co-infections of novel and seasonal influenza, thereby potentially lessening the severity of the clinical course of illness and reducing the risk of reassortants. Population-specific health communication may be effective to disseminate these public health measures to at-risk populations ((27)).

Global surveillance of HPAI A(H5N6) and a OneHealth approach are recommended to detect virological, epidemiological, and clinical changes that can affect both animal and human health. As this pathogen continues to circulate in bird populations and contaminate various environments, additional detections of sporadic human cases of A(H5N6) are to be expected. Timely information sharing of these cases and relevant clinical, epidemiological, and virological findings under the International Health Regulations (2005) remains key for human A(H5N6) infection risk assessment and mitigation ((21)). Comprehensive data sharing is necessary for capturing a true picture of human A(H5N6) cases and the risks leading to infection. Only then will public health officials be able to implement protective measures that target those at increased risk of infection, illness, and death. The maintenance of a minimum dataset of International Health Regulations (2005) notified events could contribute positively to comprehensive data sharing and effective surveillance. Collection of basic epidemiological information on the A(H5N6) cases in this study required the triangulation of multiple event-based and official reports, genomic data banks, and research publications, which is an inefficient way to maintain essential situational awareness and to inform risk assessments.

## Strengths and limitations

Although every effort was made to utilize valid and as complete as possible data element information on all cases, this study was limited by reliance on disseminated information from both official and unofficial sources. Incomplete data were often provided, with missing information for variables such as exposure history, comorbidities, or final outcome. The timing, types of case information, and the reporting formats that were shared varied widely from case to case, even in those reported by official sources. Analyses involving these data elements must thus be interpreted with caution due to the potential of demonstrating skewed population characteristics.

The A(H5N6) disease severity, highlighted by a relatively high CFR (39%) In humans, should be interpreted with caution as well, as this percentage may be subject to bias introduced by underreporting. It is possible that cases are tested more often when severe or hospitalized, and as a result of which a higher proportion of hospitalizations may be reported. In this scenario, an overestimation of the CFR in humans may occur, since the denominator might not capture mild or asymptomatic cases. It is also possible that community deaths are undercounted/underreported, such as instances in which individuals do not present to hospital and are not tested for AIV infections. However, current evidence suggests mild or asymptomatic illness is uncommon with A(H5N6), and less likely to occur as compared with other AIVs, such as A(H9N2) ((19,28,29)).

This study presented several strengths. For one, the information was gathered quickly due to the use of already published reports and articles; study authors did not need to wait for more sporadic cases to emerge to collate and analyze data. In addition, maintenance of an ongoing surveillance system (the IMAT), in which study authors collated daily respiratory events and created monthly reports on target pathogens, also supported the data collection stage. Conducting this study also highlighted the importance of not only sharing information in the international context, but also sharing complete information. Too often, case reports leave out several pertinent details about the case, resulting in potential misrepresentation of the susceptible population. However, case information was updated by study authors as more information became known through the maintenance of the IMAT, which supported the descriptive analyses, since the most complete case information possible was used.

## Conclusion

This study contributes to the existing evidence base by providing an epidemiologic analysis of all human cases of A(H5N6) with symptom onset between February 2014 and June 30, 2023, to facilitate better understanding of the characteristics of these cases. Awareness of susceptible populations is vital in informing public health measures, such as public health communication and targeted communication to populations at increased risk of infection and/or severe outcomes. With an increased incidence of human A(H5N6) cases in recent years and a disease spectrum that includes severe disease or death, surveillance and timely and complete information sharing of human cases of A(H5N6) is critical for human A(H5N6) infection risk assessment and mitigation.
